# Effect of collagenase–gelatinase ratio on the mechanical properties of a collagen fibril: a combined Monte Carlo–molecular dynamics study

**DOI:** 10.1007/s10237-019-01178-6

**Published:** 2019-06-03

**Authors:** Bethany Powell, David C Malaspina, Igal Szleifer, Yasin Dhaher

**Affiliations:** 1 Department of Mechanical Engineering and Bioengineering, Valparaiso University, Valparaiso, IN USA; 2 Department of Biomedical Engineering, Northwestern University, Evanston, IL USA; 3 Institut de Ciencia de Materials de Barcelona (ICMAB-CSIC), Campus UAB, Bellaterra, Barcelona Spain; 4 Department of Chemistry, Northwestern University, Evanston, IL USA; 5 Department of Chemical and Biological Engineering, Northwestern University, Evanston, IL USA; 6 Department of Physical Medicine and Rehabilitation, University of Texas Southwestern, Dallas, TX USA; 7 Department of Orthopedic Surgery, University of Texas Southwestern, Dallas, TX USA

**Keywords:** Collagen fibril, Mechanical properties, Collagenase, Gelatinase, Molecular dynamics, Monte Carlo

## Abstract

Loading in cartilage is supported primarily by fibrillar collagen, and damage will impair the function of the tissue, leading to pathologies such as osteoarthritis. Damage is initiated by two types of matrix metalloproteinases, collagenase and gelatinase, that cleave and denature the collagen fibrils in the tissue. Experimental and modeling studies have revealed insights into the individual contributions of these two types of MMPs, as well as the mechanical response of intact fibrils and fibrils that have experienced random surface degradation. However, no research has comprehensively examined the combined influences of collagenases and gelatinases on collagen degradation nor studied the mechanical consequences of biological degradation of collagen fibrils. Such preclinical examinations are required to gain insights into understanding, treating, and preventing degradation-related cartilage pathology. To develop these insights, we use sequential Monte Carlo and molecular dynamics simulations to probe the effect of enzymatic degradation on the structure and mechanics of a single collagen fibril. We find that the mechanical response depends on the ratio of collagenase to gelatinase—not just the amount of lost fibril mass—and we provide a possible mechanism underlying this phenomenon. Overall, by characterizing the combined influences of collagenases and gelatinases on fibril degradation and mechanics at the preclinical research stage, we gain insights that may facilitate the development of targeted interventions to prevent the damage and loss of mechanical integrity that can lead to cartilage pathology.

**Electronic supplementary material:**

The online version of this article (10.1007/s10237-019-01178-6) contains supplementary material, which is available to authorized users.

## Introduction

In healthy tissue, collagen production is typically balanced by collagen degradation mediated by matrix metalloproteinases (MMPs), enzymes that bind and cleave the triple helical collagen molecule. This balance is disrupted in many disease states, including arthritis, cancer, and fibrosis (Cawston and Wilson 2006; Clark et al. 2008; Giannandrea and Parks 2014; Malemud 2006; Mancini and Di Battista 2006). When the balance favors destruction, permanent structural changes can occur, especially in tissues that are metabolically mostly quiescent, such as articular cartilage (Maroudas et al. 1992; Verzijl et al. 2000). Such damage can lead to changes in mechanical properties that impair the function of the tissue.

At the molecular level, collagen degradation is mediated by two MMPs subfamilies: collagenases and gelatinases. Single-molecule tracking techniques reveal that collagenases (MMP-1) adsorb on collagen and then spend the majority of their time in paused states where cleavage eventually occurs (Sarkar et al. 2012). Once collagenase has cleaved a tropocollagen molecule, the tropocollagen begins to denature and becomes susceptible to gelatinase cleavage. Employing a single-molecule tracking paradigm, Rosenblum et al. (2010) reported that gelatinase (MMP-9) does not efficiently bind to the native triple helical structure, but tends to bind and cleave the partially denatured fragments that collagenases create. While the isolated interactions of MMPs with their substrates have been experimentally characterized, the combined influences of collagenase and gelatinase on the structural and mechanical properties of an isolated collagen fibril have not been established.

Several experimental examinations have shown that collagen degradation mediated by MMP subtypes produces a dramatic decrease in the mechanical strength and toughness of collagen fibers (Laasanen et al. 2003; Panwar et al. 2013, 2015; Park et al. 2008). But, while connections between the molecular structure and the strength and toughness of collagen have been drawn in some of these studies (Panwar et al. 2013, 2015), the precise nature of the relationship between MMP-induced structural changes and the mechanical properties of collagen fibrils has not been comprehensively studied. In our recent work (Malaspina et al. 2017), we employed a coarse-grained molecular dynamics (MD) model (Depalle et al. 2015) to examine how random surface degradation influenced the mechanical response of a collagen fibril. We showed that the surface degradation (determined using a solvent-accessible surface area constraint) produced dramatic changes in the toughness of the collagen fibril consistent with experimental observations (Laasanen et al. 2003; Panwar et al. 2013, 2015; Park et al. 2008). These findings align with the hypothesis that the molecular organization of the fibril exerts a strong influence over the mechanical strength (Fratzl 2008) and a small disruption of the structure leads to a large reduction of the fibril toughness. However, although the model yielded important insights into the relationship between degraded fibril structure and mechanical properties, our previous work did not account for the complexity of structural changes that may stem from the underlying biology and the thermodynamics of degradation, which includes the combined action of the two MMP subtypes, collagenase and gelatinase.

Accordingly, our purpose is to examine the combined effect of collagenase and gelatinase on collagen fibril structure and obtain the associated changes in the mechanical properties using molecular simulations. To do so, we developed a Metropolis Monte Carlo (MC) approach to examine the process of biological degradation of a collagen fibril by collagenase MMP-1 and gelatinase MMP-9. The structures obtained by the MC approach were then used as inputs to the coarse-grained MD model (Malaspina et al. 2017) to examine the changes in the mechanical response of the fibril that resulted from MMP-induced structural changes. We hypothesized that a higher ratio of collagenases to gelatinases would lead to reduced toughness of the fibril at a fixed amount of degradation, since an increase in the number of collagenases would increase the number of cleaved bonds that cannot bear load. Further, we examined the time dependence of degradation, hypothesizing that the time course of degradation would differ based on the absolute number of collagenases and gelatinases on the fibril as well as the ratio of collagenases to gelatinases. To our knowledge, this is the first study to examine the relative influences of collagenases and gelatinases acting simultaneously on a collagen fibril and the first study to quantify their relative effects on fibril mechanical integrity. We argue that this computational construct will help inform future experimental designs and consequently serve as a bridge between experimental observations and molecular mechanisms. Our results demonstrate that the ratio of collagenase to gelatinase dictates fibril degradation and mechanical integrity. The findings presented herein could eventually inform future experiments that may provide targeted clinical treatment strategies, which would likely hinge on the relative effects of local collagenase and gelatinase inhibition on degradation and mechanics.

## Methods

### Dynamic Metropolis Monte Carlo Degradation Model

**Fig. 1:**
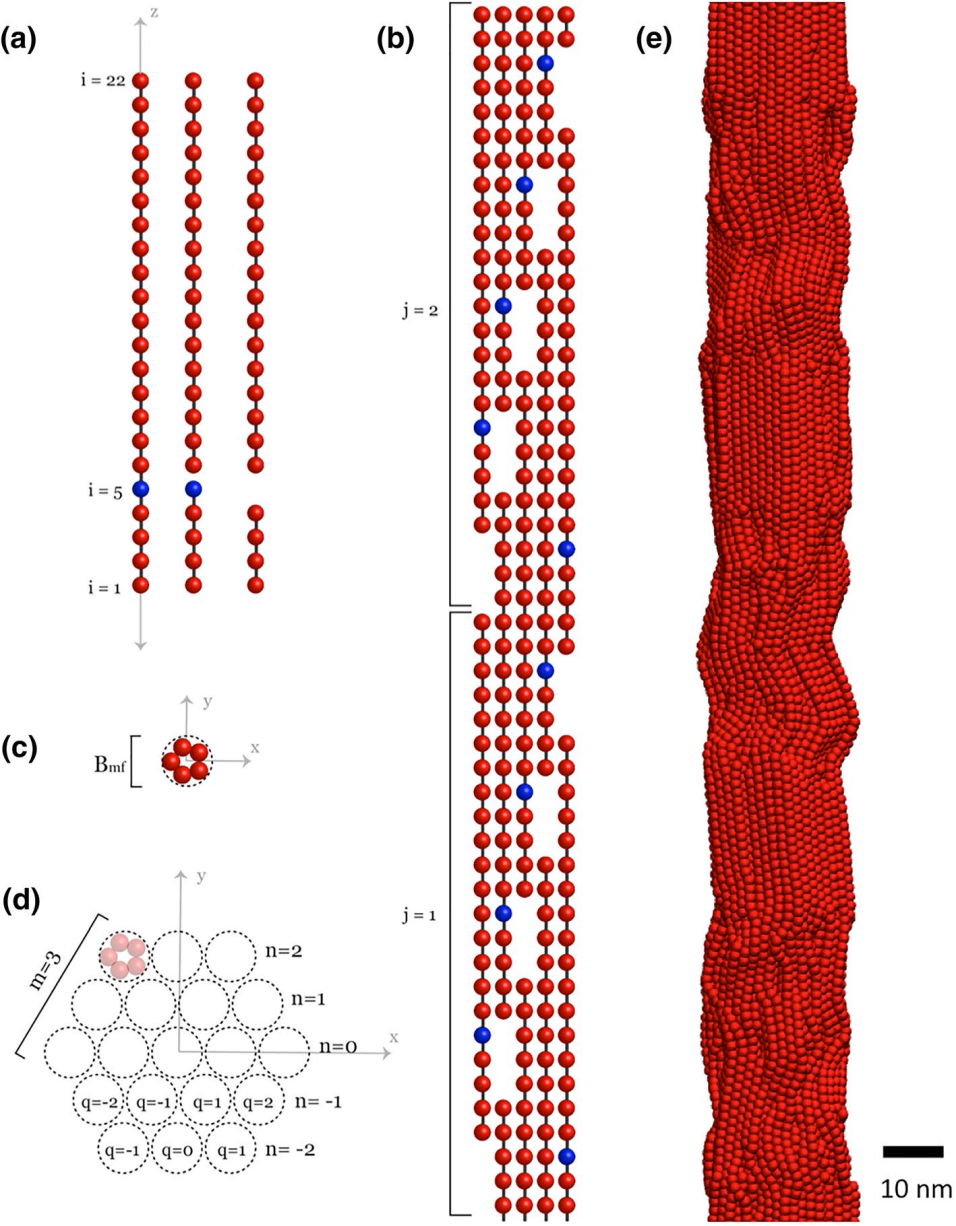
Illustrations of the components of the fibril MC lattice structure and resulting MD structure. **a** Illustration of the individual tropocollagen molecule. An intact molecule is shown on the far left, a molecule that has been cleaved at the collagenase cleavage site is shown in the middle, and a molecule with a removed lattice site is shown on the far right. The blue site denotes the position where collagenase binds with high affinity and eventually cleaves. **b** Illustration of the microfibril structure (length-wise). The pattern of gaps and staggering of the tropocollagen molecules is shown in this flattened depiction. The index j indicates the vertical position of an individual tropocollagen within a microfibril. **c** Illustration of the microfibril cross section. $B_{\text{mf}}$ is the diameter of a single microfibril. **d** Illustration of the fibril cross section when *m* = 3 (in the simulations presented in the results, *m* = 4). The index m is the number of microfibrils per edge on a fibril, and n and q are indices of microfibril position within the fibril. Refer to S1 Text for more details on the indices used to describe the structure. **e** Snapshot of a section of the fibril equilibrated by MD simulation. The cleaved and removed sites were obtained from the results of the MC simulations

A coarse-grained lattice model was used to represent the structural hierarchy of collagen, which is depicted in Fig. 1. Enzymes were randomly selected and placed on empty lattice sites using random selection of position indices on the surface of the fibril. A detailed description of the structure and the initial placement of the enzymes can be found in S1 Text. Briefly, the lattice was organized according to experimental reports of collagen fibril organization, which includes tropocollagen molecules arranged into microfibrils and microfibrils arranged into a fibril. This structure served as the basis for a dynamic Metropolis MC approach, depicted in Fig. 1a–d.

#### MMP–Collagen Interaction Energies

When an MMP occupied a lattice site on a given tropocollagen, it bound to the site with an energy that depended on the type of MMP, its position on the tropocollagen, and whether the tropocollagen molecule had been cleaved by a collagenase. The interaction energy between collagenase and the collagen lattice sites was described by

1 $$E_{{{\text{C}},{\text{colla}}}} = \left\{ {\begin{array}{*{20}l} {0,} \hfill & {i \le 4} \hfill \\ { - 2kT,} \hfill & {i = 5} \hfill \\ {0,} \hfill & {i > 5} \hfill \\ \end{array} } \right.$$

where the first subscript *C* denotes the enzyme type, collagenase, the second subscript colla denotes the substrate, collagen, *k* is the Boltzmann constant, *T* is the temperature, and *i* is the index of vertical position along a tropocollagen. These interaction energies were estimated by running the MC model with a single collagenase on the fibril, calculating the percent of steps where the collagenase was paused at its characteristic cleave site (*i* = 5). The energy was adjusted until the collagenase remained at the characteristic cleave site during approximately 18% of the total simulation steps, since Sarkar et al. (2012) observed that collagenases adsorbed to a fibril spend 18% of their time paused at exposed collagenase cleave sites, which they denoted as “class II pauses.” Further, experiments indicate that collagenases are unable to diffuse over cleaved sites and have lower affinity for gelatin (Sarkar et al. 2012; Saffarian et al. 2004); therefore,

2 $$\begin{aligned} E_{{{\text{C}},{\text{Ccl}}}} & = \infty \\ E_{{{\text{C}},{\text{gela}}}} & = 0.5\,kT, \quad 1 \le i \le 22 \\ \end{aligned}$$

where *C* denotes collagenase, Ccl denotes a cleaved lattice site, and the subscript gela denotes gelatin (a tropocollagen molecule with at least one cleaved site). While the exact change in collagenase affinity for collagen after cleavage is unknown, we incorporated an increase in the interaction energy to phenomenologically incorporate qualitatively observed collagenase behavior.

Gelatinase interactions with collagen and gelatin, respectively, were described by the equations

3 $$\begin{aligned} E_{{{\text{G}},{\text{colla}}}} & = 0, \quad 1 \le i \le 22 \\ E_{{{\text{G}},{\text{gela}}}} & = - 0.5\,kT, \quad 1 \le i \le 22 \\ \end{aligned}$$

where the subscript *G* indicates gelatinase, colla denote an uncleaved tropocollagen, and the subscript gela indicates a cleaved tropocollagen or gelatin. Again, the exact change in the gelatinase affinity for collagen after cleavage is unknown, but we incorporated a decrease in the interaction energy to phenomenologically take experimental observations into account (Rosenblum et al. 2010).

#### Dynamic Metropolis Monte Carlo Moves

A dynamic Metropolis MC approach was used to simulate the interactions between the MMPs and the collagen to capture the stochastic, time-dependent nature of the collagen degradation process.

For each step of the simulation, an enzyme was randomly selected from the ensemble of collagenases and gelatinases, and the selected enzyme attempted to move vertically or horizontally or attempted to cleave. The movement attempts were accepted based on the Boltzmann factor of the change in energy between one lattice site and the next.

For vertical motion, trial moves in the positive and negative *z*-directions were chosen with the same probability. In a similar way, trial moves were chosen with the same probability for “left or right” movements for horizontal motion but constrained to remain on the outermost edge of the fibril. In accordance with experimental observations (Sarkar et al. 2012), the ratio of vertical to horizontal trial moves was selected in the model such that vertical movements were much more probable. Furthermore, the probabilities of displacement attempts were selected such that the enzymes would move with the diffusion coefficients reported in the literature (Collier et al. 2011).

For cleave attempts, the model presented here was parameterized to mimic experimental observations (Sarkar et al. 2012; Rosenblum et al. 2010). Collagenase cleavage only occurred when collagenases were positioned on the lattice site 66 nm from the C terminus of an individual tropocollagen (*i* = 4), and gelatinase cleavage only occurred when gelatinases were positioned on a tropocollagen that had already been cleaved by a collagenase (Rosenblum et al. 2010; Fields 2013). When the enzymes were in the positions where cleavage could occur, cleavage succeeded at an overall rate of 0.35 s^−1^ (Sarkar et al. 2012). When cleavage occurred, a bond was broken between the N terminus of the occupied site and the C terminus of the site directly above the occupied site (Fig. 1a).

Once a lattice site and its lower neighbor had been cleaved, that site became disconnected from the rest of the lattice and was removed from the system immediately (Fig. 1a). As the removal progressed, some sets of lattice sites became detached from neighboring lattice sites and were removed from the system. A set of lattice sites was considered detached when it had no vertical neighbors, and when it was not attached to at least two other beads horizontally.

A semi-quantitative verification of the MC simulations of the degradation process against experimental observations are shown in S1 Fig. Furthermore, S1 Text includes a detailed description of the MC model formulation.

#### Degradation Conditions

The number of enzymes on the fibril was selected in a way that facilitated study of the relative effects of collagenases and gelatinases, since the patterns of cleavage differ for the two classes of MMPs. The amount of each type enzyme can be found in Table 1. For each enzyme surface coverage condition, the changes in fibril structure were recorded as the simulation progressed.

**Table 1:** Simulated systems and relative amounts of collagenases and gelatinases

Simulated system	Number of collagenases	Number of gelatinases	Ratio of collagenase to gelatinase
System 1 (c 4: g 4)	4	4	1
System 2 (c 8: g 8)	8	8	1
System 3 (c 6: g 2)	6	2	3
System 4 (c 2: g 6)	2	6	0.33

### Mechanical Analysis with Coarse-Grained Molecular Dynamics

The degraded structures from the MC simulations were then used to perform mechanical analysis using MD. The mapping of sites from the lattice MC to the MD model (Malaspina et al. 2017) is such that one site on the MC structure is equivalent to ten sites in MD structure. The simulation protocol is identical to the one used in our previous work (Malaspina et al. 2017) with a fibril that is ten times longer. The length was increased so that the size of the MD structure would be equal to the MC structure. In the MD structure, which is depicted in Fig. 1e, each tropocollagen is represented by a polymeric chain that contains 220 bonded beads. A total of 925 tropocollagen chains interact through a Lennard–Jones potential, in conjunction with a harmonic potential and a hyper-elastic bond energy. The force field for the coarse-grained MD model was developed by Depalle et al. (2015) and is described in more detail in S2 Text.

The stress–strain response was assessed at 1.1% degradation for all treatment conditions, where percent degradation was defined as number of removed lattice sites divided by the initial number of lattice sites × 100. This amount of degradation (1.1%) was chosen based on a previous work (Malaspina et al. 2017) and represents the beginning of a plateau in the change in toughness of a collagen fibril as function of surface degradation. Since the MC degradation simulation progressed at different rates for each condition, the time required to reach 1.1% degradation differed for each system described in Table 1. Regardless of the time required to reach 1.1% degradation, the analysis was performed at a single instant in time, and no further enzyme-mediated degradation occurred as the tensile loading was applied.

For the quantification of the fibril stress–strain response to axial loading, the strain was computed by tracking the change in the size of the simulation box and the stress tensor was computed using the virial stress in the MD package LAMMPS (Plimpton 1995). The strain rate used in these simulations was $10^{7} \,{\text{s}}^{ - 1}$. In the current model, the abrupt fracture of the fibril does not occur because the fracture energy dissipation was constrained with a maximum velocity limit to avoid the collapse of the simulation at large strains.

We analyzed the strain distribution inside the fibril by calculating the tropocollagen bond length distributions. The distribution is calculated based on the length of bonds in the fibril structure at the different fibril bulk strain values. This distribution allows for the quantification of sliding and stretching of tropocollagen chains during the deformation of the fibril. We defined bonds as sliding bonds when their length during fibril deformation was comparable to their length in the undeformed fibril, and we defined bonds as stretching bonds when their length during fibril deformation was large compared to their length in the unstretched fibril.

## Results

### Fibril Morphology After Degradation

**Fig. 2:**
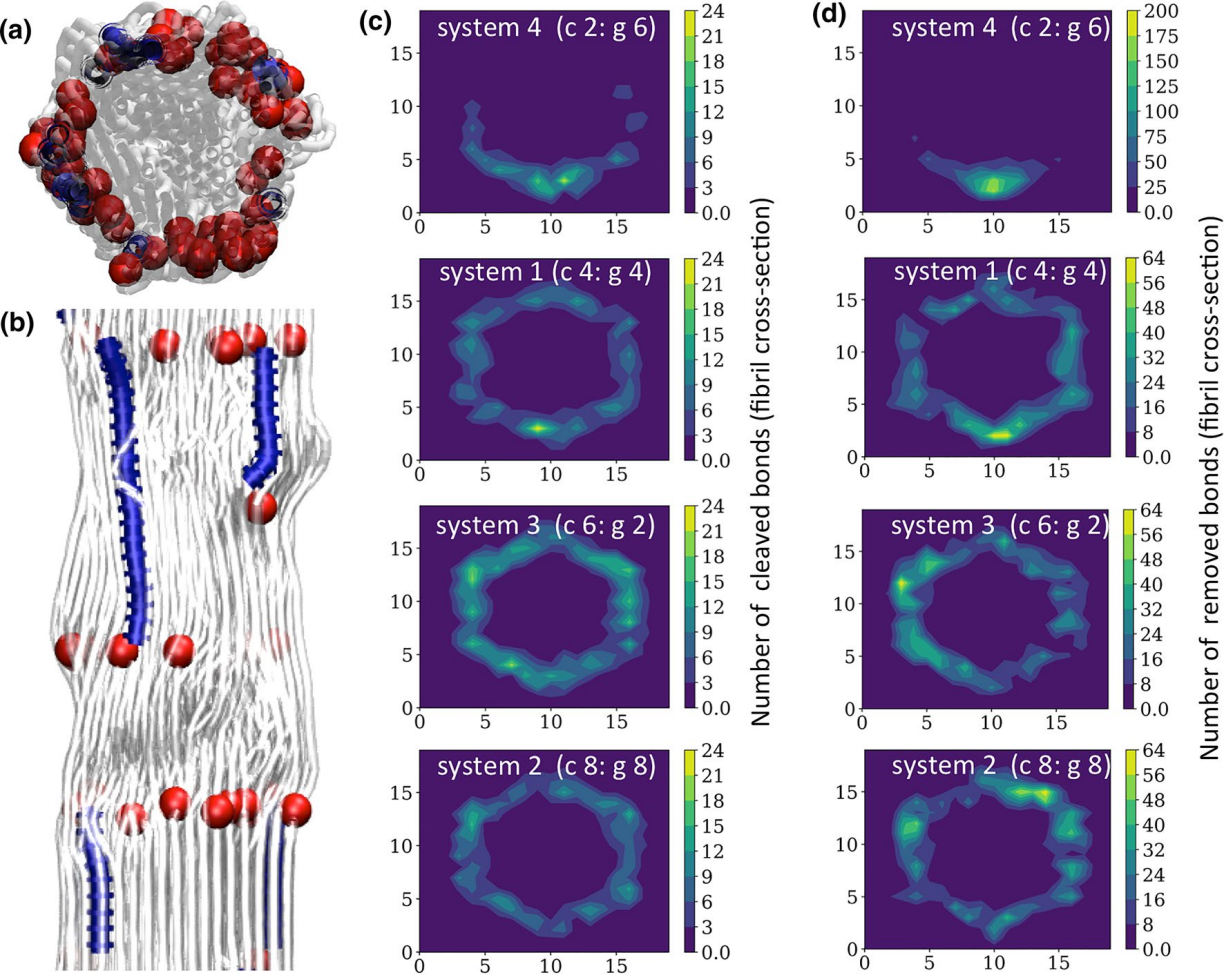
Representation of cleaved and removed sites for different conditions. **a** Snapshot of a cross-sectional view of the fibril and **b** longitudinal view of the fibril. In **a**, **b** removed sites are represented as blue tubes and cleaved sites are represented as red spheres. **c** Density map of cleaved sites from the fibril cross-sectional view for system 4, 1, 3 and 2, ordered from top to bottom, respectively. **d** Density map of removed sites from the fibril cross-sectional view for system 4, 1, 3 and 2, ordered from top to bottom, respectively

We sought to study the combined effects of collagenases and gelatinases on fibril degradation, and the subsequent changes to the mechanical properties of collagen fibrils. To do so, we used a combined MC/MD approach and observed complex changes to the fibril structure when collagenases and gelatinases degrade a collagen fibril simultaneously. The MC model revealed distinct distributions of cleaved and removed beads along the fibril for structures that had all experienced 1.1% degradation (Fig. 2). A snapshot of a section of an equilibrated fibril (Fig. 2a, b gray), displaying removed beads (Fig. 2a, b blue lines) and the beads with adjacent cleaved bonds (Fig. 2a, b red spheres), served as an illustration of the general distribution of cleaved and removed bonds obtained from the MC simulation. Note that in the current study, one removed site in the MC simulation was equivalent to 10 sites in the MD simulation, which is why the removed sites were represented by blue lines instead of individual beads in the MD snapshot (Fig. 2b). The spatial distribution of cleaved sites from the cross-sectional view of the fibril for all the systems can be observed in the heat maps of Fig. 2c. In a similar way, the spatial distribution of removed sites from the cross-sectional view of the fibril for all the systems can be observed in Fig. 2d. In both Fig. 2c and Fig. 2d, the heat maps are purposely arranged the lowest amount of collagenase (top panels) to the highest amount of collagenase (bottom panels) to better illustrate the behavior of the systems.

The density and location of cleaved bonds were highly dependent on the ratio of collagenase to gelatinase, as indicated by the heat maps in Fig. 2c. System 4 (c 2: 6 g, top panel Fig. 2c) had the smallest ratio collagenase to gelatinase and the most localization of cleaved bonds. As the ratio of collagenase to gelatinase increased, the distribution of cleaved bonds became more uniform across the outer surface of the fibril cross section, as shown in System 1 (4 c: 4 g, second panel Fig. 2c). As the ratio of collagenase to gelatinase continued to increase, the density of cleaved bonds increased around the outer surface of the fibril cross section, as shown in System 3 (6 c: 2 g, third panel Fig. 2c). Finally, System 2 (8 c: 8 g, bottom panel Fig. 2c) showed a similar distribution and density of cleaved bonds compared to System 1 (4 c: 4 g, second panel Fig. 2c), which had the same ratio of collagenase to gelatinase as System 2 but half the total number of enzymes. This observation indicates that, for the same amount of degradation, the fibril had less connectivity (more cleaved bonds) on the surface as the ratio of collagenase to gelatinase increased. Moreover, for a small ratio of collagenase to gelatinase, the distribution of cleaved sites tended to be more localized.

The density map of removed beads (Fig. 2d) appeared to be related to the density map of cleaved bonds (Fig. 2c), since gelatinase could remove a bead in the MC model only if a collagenase had previously cleaved a neighboring bond. Thus, the location of removed bonds (Fig. 2d) was similar to the density map of cleaved bonds (Fig. 2c) but much more localized in certain regions. Note that the number of removed beads was the same for each of the panels in Fig. 2d, but the distribution of the removed beads differed for each system. Moreover, the distribution appeared to differ for a small ratio of collagenase to gelatinase (see system 4; c 2: 6 g, top panel Fig. 2d), but the effect of the collagenase to gelatinase ratio was less evident in the other systems (Fig. 2d, bottom 3 panels).

The time course of degradation appeared to depend on the ratio of collagenases to gelatinases, as shown in S1 Fig. Degradation progressed most quickly when more gelatinases were present (system 4) and progressed most slowly when more collagenases were present (system 3) when the total number of enzymes on the surface was constant. However, when the absolute number of enzymes on the fibril was increased, the degradation progressed more quickly (system 2).

### Toughness of the Fibril

**Fig. 3:**
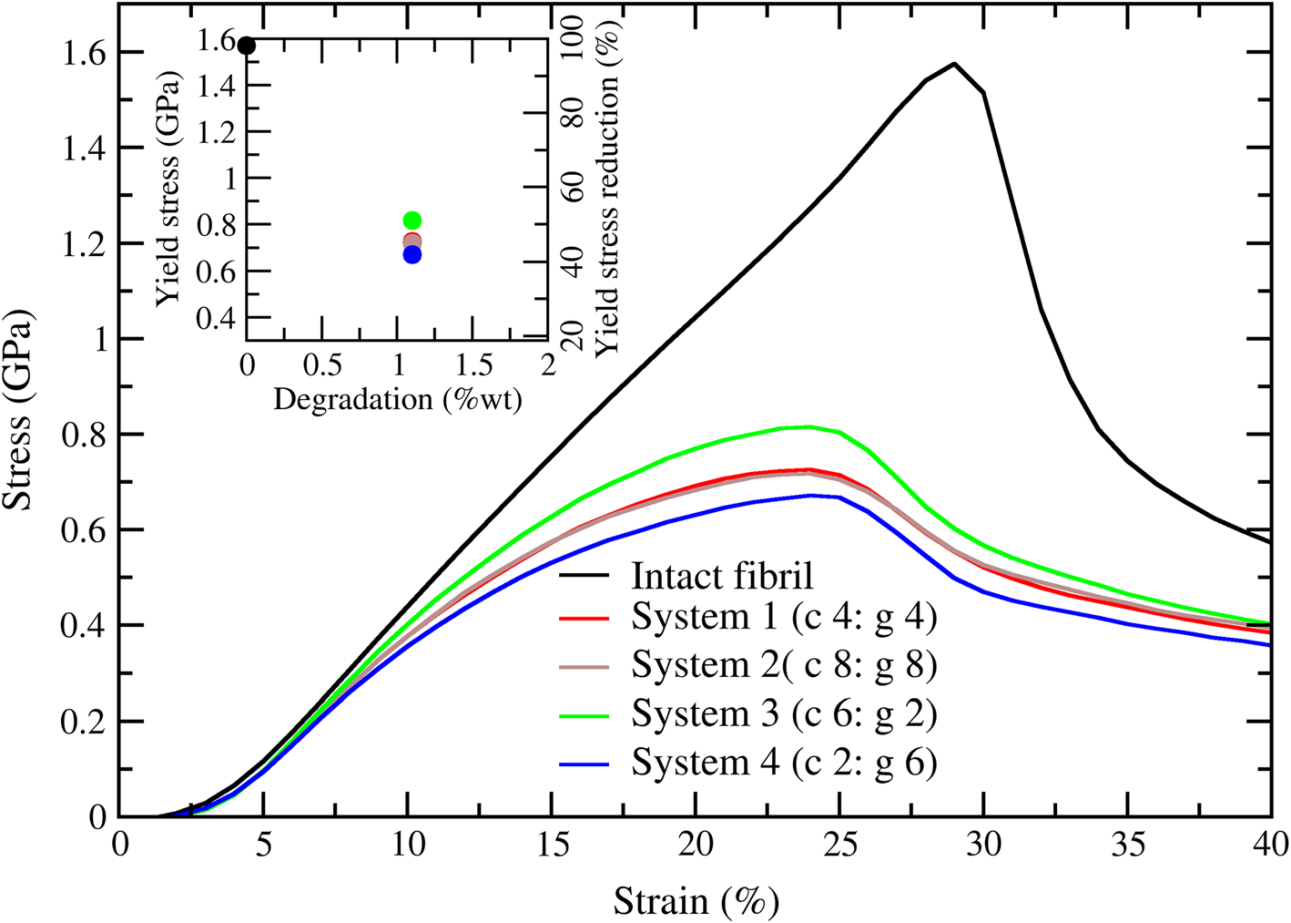
Stress–strain curves for different collagenase and gelatinase amounts at 1.1% degradation. Simulated systems 1, 3 and 4 are represented in solid lines and system 2 is represented in blue dashed line to denote difference in total amount of enzymes. The stress–strain curve for the intact fibril is included in gray dashed lines

To analyze the simultaneous effect of degradation by collagenase and gelatinase on the mechanical properties, we studied the stress–strain response by using MD simulations. The stress–strain curves are shown in Fig. 3 for the simulated systems in Table 1. As a reference, we included the stress–strain behavior of an intact fibril with its characteristic deformation regions (Malaspina et al. 2017; Depalle et al. 2015). The fibril exhibited an abrupt change in the mechanical properties as a result of small amount of degradation and showed a shift in the yield strain for the degraded curves, characteristics consistent with our earlier report (Malaspina et al. 2017). One key observation is that the fibril toughness was progressively reduced as the collagenase-to-gelatinase ratio increased. Moreover, the stress–strain curves for a ratio of 1 (systems 1 and 2) were nearly identical, even though the absolute numbers of enzymes differed by a factor of two, indicating the stress–strain curves for a specified level of degradation appeared to depend on the ratio of collagenases to gelatinases and not the total number of enzymes. The yield stress was reduced by ~ 52% for the system with the smallest ratio of collagenase to gelatinase (system 4) and ~ 43% for the system with the largest ratio collagenase to gelatinase (system 3; Fig. 3 inset).

### Bond Length Distribution at Fixed Strain

**Fig. 4:**
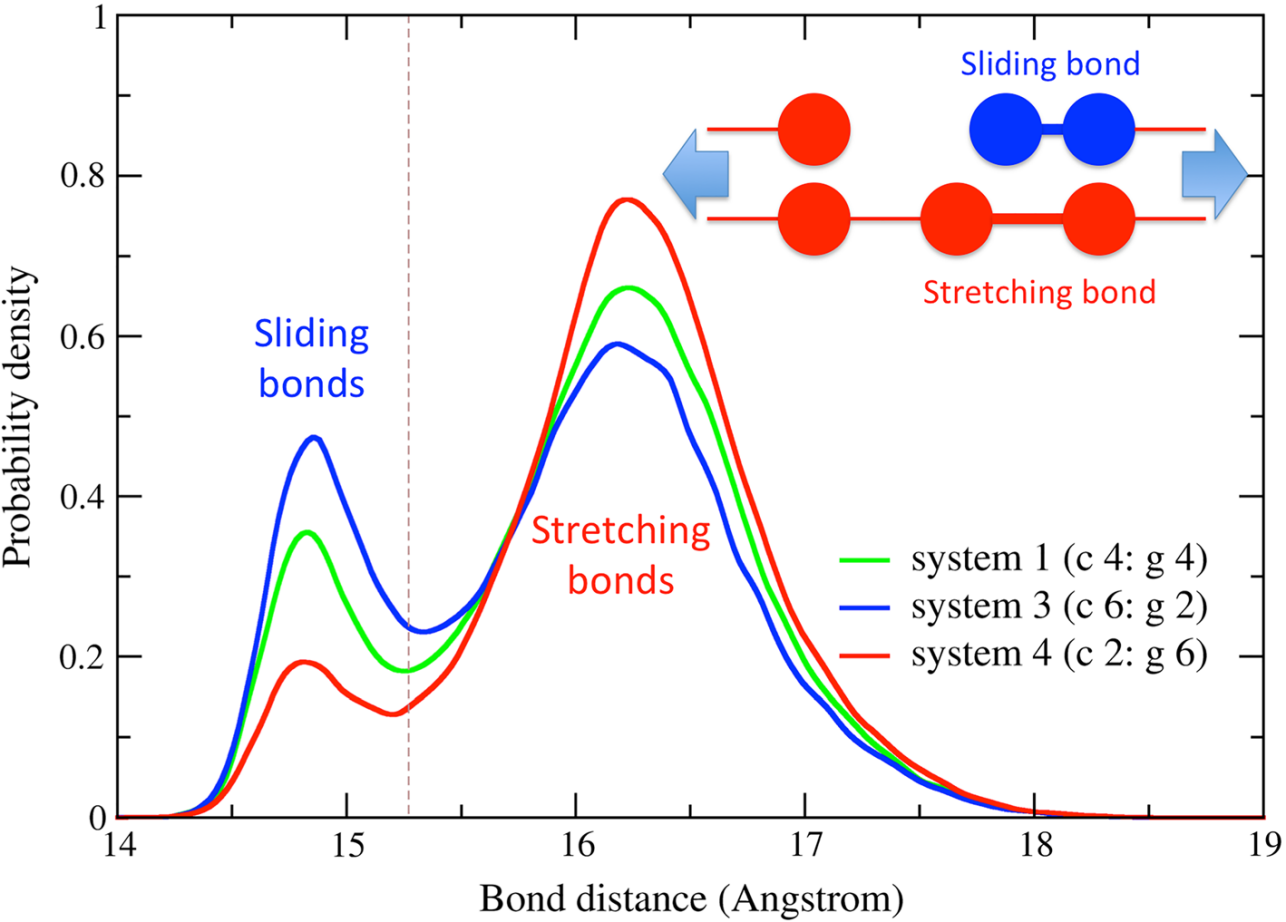
Distributions of tropocollagen bond lengths for systems 1, 3 and 4 described in Table 1. The distribution is evaluated at the same strain (19%) and same degradation (1.1 wt%). A gray dashed line represents the mean value where bonds transition from sliding to stretching. An inset scheme is included to show the difference between sliding and stretching bonds

Bond length distribution was examined to investigate the molecular origin of the change in toughness for the different stress–strain curves in Fig. 3. These distributions provided the probabilities of tropocollagen stretching and tropocollagen sliding (Fig. 4 inset schematic) and were related to the stress distribution inside the fibril. Two populations of bond lengths could be observed for each structure when the strain was equal to 19%, a pre-yield state (Fig. 4). The probability peak associated with the lower lengths was centered slightly above the equilibrium bond length of 14.7 Angstroms, indicating that minimal stretching had occurred and that the beads connected by those bonds were sliding with respect to the rest of the structure. The peak centered around higher lengths was associated with stretching of the bonds, indicating that the bonds connecting these beads could bear load (Malaspina et al. 2017). As the collagenase-to-gelatinase ratio increased, the proportion of unstretched (sliding) bonds increased and the proportion of stretched bonds decreased. This trend suggested that fewer tropocollagen molecules bore load and more molecules passively slid apart as the ratio increased, which may explain why fibril toughness decreased as ratio increased.

### Bond Length Distribution Versus Strain

**Fig. 5:**
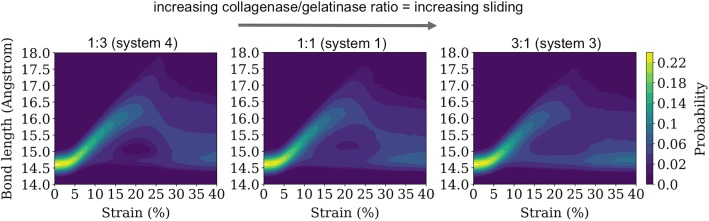
Contour plot of the distribution of bond lengths as a function of the strain for a collagen fibril at different collagenase/gelatinase ratios. The ratios are indicated above the plots, which are arranged in increasing ratio collagenase/gelatinase from left to right

The analysis of the bond length distribution was extended to the whole range of strains calculated in Fig. 3. The distribution of bond lengths is displayed in Fig. 5 as a contour plot to identify how the distribution of bond lengths evolved as the fibril stretched. At low strains, the bond length distributions showed that almost all of the bonds were sliding bonds. As the strain increased, a bimodality in the distribution started to develop, representing both stretching and sliding bonds. This bimodality can be identified in Fig. 5 as the region containing a “hole” (dark blue) that represents the minimum between the sliding and stretching peaks (also shown in Fig. 4). At midrange strains, system 3 appeared to show this bimodality, while systems 1 and 4 did not appear to have as many sliding bonds in this range of strains before yield. However, as the strain increased beyond yield, the distributions of bond lengths shifted back toward the equilibrium bond length of 14.7 Angstroms for all three systems.

## Discussion

### Lessons Learned

Using sequential MC and MD simulations, we probed the effect of enzymatic degradation on the structure and mechanics of a single collagen fibril. These models investigated the combined degradation behavior of collagenase and gelatinase and the molecular origin of the mechanical integrity of a degraded collagen fibril. Our MD simulations revealed that the loss of fibril mechanical integrity may depend not only on the number of removed beads, but also on the relative abundance of collagenase and gelatinase. An increase in the amount of collagenase increased the total number of cleaved bonds at a fixed amount of degradation. Further, the ratio of collagenase to gelatinase appeared to regulate the spatial distribution of the cleaved sites on the fibril surface, with a more diffuse pattern of cleaved bonds for higher ratios and a more localized pattern for low ratios. Finally, our examination of the bond length distribution demonstrated that the fibrils with reduced toughness had fewer stretched bonds and more sliding bonds compared to the tougher fibrils. These results, which demonstrated a complex interplay between collagenase and gelatinase effects on fibril degradation and mechanical integrity, could inform future experiments that would provide targeted clinical treatment strategies, which would likely hinge on the relative effects of local collagenase and gelatinase inhibition on degradation and mechanics.

The toughness of the fibril appeared to be linked to the connectivity of the fibril, which we characterized using bond length distributions (Fig. 5). These distributions revealed that increases in the relative amount of collagenase led to more sliding bonds and fewer stretched bonds, which may help explain the reduced load-bearing capacity of fibrils that were exposed to higher ratios of collagenase to gelatinase. The sliding bonds only provided a small amount of resistance to fibril loading through non-bonded cohesive molecular forces within the fibril (hydrogen bonds, electrostatic and van der Waals interactions). However, the stretched bonds provided greater resistance to load because they were connected by covalent bonds in addition to the cohesive molecular forces within the fibril. Thus, because sliding bonds indicate reduced load-bearing capacity, the presence of more sliding bonds (shown in Fig. 5) may help explain the overall reduction in toughness that was observed for systems with more collagenases (shown in Fig. 3).

Our simulation results indicated that the relative abundance of collagenases and gelatinases affected the spatial distribution of fibril degradation (Fig. 2c, d). It appeared that a low ratio would lead to damage that was more localized to one region of the fibril surface, while a higher ratio would lead to a more diffuse pattern of cleaved bonds and removed fragments. For lower ratios, the damage remained confined to a particular region of the fibril, which may have allowed more bonds in the undamaged regions to stretch and bear the applied load. Conversely, the diffuse pattern of cleaved sites may have disrupted the continuity of the molecular structure at more locations along the fibril surface and prevented the uptake of load by the individual tropocollagen molecules. Thus, multiple lines of evidence seem to help explain the observed differences in load-bearing capabilities for the systems with different ratios of collagenase to gelatinase (Fig. 3).

Although the mechanical response of a fibril appeared to depend on the ratio of collagenases to gelatinases and not on the absolute number of enzymes after a fixed amount of degradation, the speed of degradation appeared to depend on both the absolute number of enzymes on the fibril and the ratio of collagenase to gelatinase. S1 Fig shows that the time course of degradation accelerated when the total number of enzymes increased (system 2) and when the collagenase-to-gelatinase ratio decreased (system 4). These two factors, MMP ratio and surface coverage, are likely regulated in vivo by local cellular production rate and clearance by the lymphatic system and may have important implications for the progression of damage in the joint. Thus, future investigations should attempt to holistically examine the progression of damage to the collagen by combining information about these two factors that influence degradation and mechanics with our understanding of the biological processes (cellular production and lymphatic clearance) that regulate the factors.

### Model Assumptions and Limitations

The MC model of degradation employed in this study included a constant number of collagenases and gelatinases on the fibril surface and did include a relationship between this surface coverage and MMP concentration in adjacent solution. That is, the model neglected the processes of adsorption and desorption. Had the model included the adsorption and desorption processes, the total number of enzymes on the fibril would have remained constant on average but would have fluctuated around that average throughout the simulation. However, it would have been difficult to include these processes in the model because the interaction energies associated with the adsorption and desorption processes have not been experimentally reported in the context of MMP–tropocollagen interactions. Thus, it would not be possible to use information about the processes of adsorption and desorption to establish a functional relationship between MMP surface coverage and MMP concentration in solution at the single fibril level. At a coarser level, kinetic studies of collagenase and gelatinase have established such functional relationships for bulk solutions. However, the mean-field approximation underlying those approaches limits their utility at the nanoscale (Collier et al. 2001; Welgus et al. 1980). Nonetheless, the MC model could be adapted to examine adsorption/desorption if the necessary parameters become available.

In the present investigation, the computational framework assumes that the entire fibril is accessible to MMPs throughout the MC simulations. We recognize, however, that a single fibril in solution does not adequately represent the environment of a fibril that is incorporated into a tissue, such as cartilage, where degradation will proceed much more slowly. Indeed, numerous reports indicate that collagen fibrils in cartilage are protected by the glycosaminoglycan (GAG) molecules that surround them (Kar et al. 2016; Li et al. 2015; Pratta et al. 2003) and that the GAGs slow the degradation process such that it occurs over years and decades rather than minutes and hours. However, this slow degradation of GAG-protected tissues can be traced back to the degradation of collagen fibrils. Thus, we argue that it is necessary to gain a fundamental understanding of single fibril degradation to fully understand the degradation at larger scales.

### Regarding Verification

Multiple lines of evidence suggest that the MD model in this work adequately reflects experimental observations. In our previous work (Malaspina et al. 2017), we compared the results of simulated mechanical testing to experimental data obtained using axial extension tests (Svensson et al. 2013). While we were only able to compare the results for intact fibrils because axial extension tests have not been performed on degraded collagen fibrils, we observed reasonable agreement between experimental and simulated results for the intact fibril. Furthermore, the MD model has been successfully applied in different scenarios (Depalle et al. 2015, 2016) where it showed general agreement with experimental observations. Finally, recent work on collagen fascicles seems to corroborate our results that show damage to the fibril will reduce its mechanical strength (Zitnay et al. 2017). That work shows that collagen fascicles subjected to sub-yield loading conditions experience subtle damage that leads to reduced strength and toughness compared to intact specimens (Zitnay et al. 2017), though we note that the mechanism of damage differs between their experiment and our model. Despite the similarities between the MD model and experimental observations, the present model, which incorporates biological mechanisms of degradation, has not yet been validated thoroughly against experimental data and further verification is needed. However, such experiments would require examinations at the single fibril level, which is experimentally challenging (Laasanen et al. 2003; Panwar et al. 2013, 2015). Some of these challenges include obtaining isolated fibrils, measuring enzyme adsorption and reaction, measuring collagen degradation, and measuring mechanical properties at the single fibril level.

A combination of approaches may be required to experimentally verify the predictions of the degradation model. Following treatment of isolated collagen fibrils with different ratios of collagenase to gelatinase, AFM imaging could be used to examine the resulting morphology of the fibrils. Indeed, high-speed AFM imaging has been used to examine degradation of collagen micro-ribbons by bacterial collagenase in real time (Watanabe-Nakayama et al. 2016). Degradation rate can also be verified by continually measuring the loss in mass of the fibril during a sustained MMPs exposure using sensitive microbalance technologies. However, these experiments may be difficult to interpret, since adsorption and desorption of the MMPs would also contribute to changes in weight. As indicated earlier, adsorption/desorption behavior of MMPs is not well understood and may be one of the key barriers in the validation of models exploring the enzyme-mediated changes in mechanical properties. While these advanced experimental approaches remain beyond the scope of the present work, the results of the model may serve as a guide to experiments that will further illuminate the combined influences of collagenase and gelatinase on the degradation of individual collagen fibrils.

### Conclusion

In summary, this study is the first step toward revealing the individual roles of collagenases and gelatinases in the structural deterioration of fibrillar collagen. The ratio of collagenase to gelatinase appears to influence the structure and, consequently, the toughness of a fibril. This knowledge of the relative roles of collagenases and gelatinases may eventually help illuminate paths to targeted treatments that may prevent or reduce pathological collagen degradation and loss of mechanical integrity, particularly in articular cartilage.

## Electronic supplementary material

Below is the link to the electronic supplementary material.

Supplementary material 1 (DOCX 28 kb)

Supplementary material 2 (DOCX 14 kb)

Supplementary material 3 (DOCX 13 kb)

S1 Fig. Degradation of collagen fibril by collagenases and gelatinases as a function of time for different collagenase to gelatinase ratios (TIFF 15952 kb)

S2 Fig. Trajectories for Collagenases Moving Along the Long Axis of the Collagen Fibril for a System with 10 Collagenases and No Gelatinases. Each curve represents the path of one of the five collagenases, showing the directional motion observed in our simulations. Though we did not include a bias toward directional motion a priori, we observed an emergent directional motion of collagenases from the C-terminus toward the N-terminus for all of the systems under investigation. z = 0 corresponds to the C-terminus of the fibril (TIFF 15909 kb)

Supplementary material 6 (DOCX 14 kb)

Supplementary material 7 (DOCX 12 kb)
