# Supplementary material for: Effect of collagenase–gelatinase ratio on the mechanical properties of a collagen fibril: a combined Monte Carlo–molecular dynamics study
Source: Biomech Model Mechanobiol. 2019 Jun 3;18(6):1809–19. doi: 10.1007/s10237-019-01178-6 (PMC6825035; doi:10.1007/s10237-019-01178-6)
Supplement: Supplementary file 1 — Supplementary material 1 (DOCX 28 kb) [file 10237_2019_1178_MOESM1_ESM.docx]

#### S1 Text: Additional details for Monte-Carlo model

#### Fibril Geometry

A coarse-grained lattice model was used to represent the structural hierarchy of collagen, which has been well characterized (21). Detailed models of the collagen fibril structure have been reported (22, 23), but we chose to use a simplified representation (Fig. 1) to reduce computational cost and facilitate the study of aggregate degradation of the fibril. Each lattice site represents a segment of collagen with a length of 14 nm and a diameter of 1.5nm. Although each site represents a volume of collagen, a lattice site, or unit, is algorithmically described as a single (x, y, z) coordinate in the center of the volume.

The (x, y, z) coordinates of each lattice site are described by the following expressions:

| $z=\left( i-1 \right)z^{*}+25\left( j-1 \right)z^{*}+5\left( h-1 \right),$  $\left\{ \begin{aligned} 1\leq i\leq22 \\ 1\leq j\leq5 \\ 1\leq h\leq5 \end{aligned} \right.$ | (1) |
| --- | --- |

| $y=b[sin \left( 72^{\circ}\left( h-1 \right) \right)+sin(r(n,q)^{\circ})]+B_{\mathrm{mf}}n\sin\left( 60^{\circ} \right),$  $-\left( m-1 \right)\leq n\leq m-1$ | (2) |
| --- | --- |

| $x=b[cos \left( 72^{\circ}\left( h-1 \right) \right)+cos(r(n,q)^{\circ})]+B_{\mathrm{mf}}q(n)$  $-[m-0.5*\left( \left\vert n \right\vert+1 \right)]\leq q(n)\leq[m-0.5*\left( \left\vert n \right\vert+1 \right)]$ | (3) |
| --- | --- |

where i, j, and h are integers representing the vertical position of a lattice site within a single tropocollagen molecule, the vertical position of a tropocollagen within a microfibril, and the cross-sectional position of a tropocollagen within the microfibril, respectively. The integers n and q(n) describe the cross-sectional positioning of a given microfibril within the fibril, specifying the row (n) and the position within row n (q(n)). The height of a single lattice site, 14 nm, was denoted as z*, and the diameters of tropocollagen molecules (1.5nm) and microfibrils (3.5nm) were denoted as b and Bmf, respectively. The random angle, r(n, q), rotates each microfibril about its long axis. A schematic of the fibril (without the random rotation of microfibrils) is depicted in Fig. 1, illustrating the indices and measurements found in Eq. 1-3.

#### Initial MMP Placement

During the initial placement, enzymes were randomly selected and placed on empty lattice sites using random selection of position indices within a microfibril (defined in Eqs. 1-3)

| $\left\{ i \right\vert1\leq i\leq22\}$  $\left\{ j \right\vert1\leq j\leq5\}$  $\left\{ h \right\vert1\leq h\leq5\}.$ | (4) |
| --- | --- |

Next, to ensure that the enzymes were placed on the outer edge, the n and q(n) indices were randomly selected such that the selected microfibril had fewer than six nearest neighbors:

| $\{(n,q\left( n \right)) \vert NN(n,q\left( n \right))<6\}$ | (5) |
| --- | --- |

where NN(n,q(n)) is the number of nearest neighbors of the selected microfibril. A nearest neighbor microfibril was defined as any microfibril whose center in the xy-plane was a distance of B_mf_ away from the center of the selected microfibril and whose cross-section contained at least two tropocollagen lattice sites at the selected z-position. Once the enzymes were placed on the surface, a fixed number of them remained on the fibril throughout the degradation simulations for the sake of computational simplicity, even though Watanabe-Nakayama et al. (24) showed collagenases adsorb and desorb on the surface of the fibril.

#### Further details of the dynamic Metropolis Monte Carlo model

A dynamic Metropolis Monte Carlo approach, depicted in Fig. 1, was used to simulate the interactions between the MMPs and the collagen to capture the stochastic, time-dependent nature of the collagen degradation process.

For each step of the simulation, an enzyme was randomly selected from the ensemble of collagenases and gelatinases, and the selected enzyme attempted to move vertically or horizontally or attempted to cleave. The movement attempts were accepted based on the Boltzmann factor of the change in energy between one lattice site and the next, defined as:

| $p=e^{\frac{-(E_{s}-E_{s-1})}{\mathrm{kT}}}$ | (6) |
| --- | --- |

where p is the acceptance probability, E_s_ is the interaction energy between the enzyme and the lattice site it occupies on step s, and E_s-1_ is the interaction energy at the previous position.

The stepwise positions of the MMPs were dictated by the equation

| $f_{s}=f_{s-1}+g,$ | (7) |
| --- | --- |

for step s of the selected enzyme where f = i and g = ±1 for vertical displacement. For vertical motion, g was chosen using a random number generator that selected values on the uniform interval [0,1). A random number in the range [0,0.5) led to a negative vertical displacement (g=-1), and a random number in the range [0.5,1) resulted in a positive vertical displacement (g=1). For horizontal motion, Eq. 10 is a vector equation where f = (n, q) and g is a two-element vector in which each element is a randomly selected integer on the interval [-1, 1]. Horizontal displacements were subject to the constraint that the selected microfibril was on the outermost edge of the fibril, as described by Eq. 5. Vertical movements were much more probable than horizontal movements, which occurred at the experimentally reported rate of 0.05 s^-1^. {Sarkar, 2012 #102}

In some instances, a vertical step would cause an enzyme to step into a gap between tropocollagen molecules due to their vertical spacing within microfibrils. For this special case, the enzyme would move to another tropocollagen molecule within the current microfibril where

| $z_{s}=z_{s-1}+g*z^{*}$ | (8) |
| --- | --- |

where z_s_ is the newly selected z-position (according to Eq. 1) and z_s-1_ is the previous z-position for the selected enzyme. Due to the shift to the new tropocollagen, i, j, and h for step s will not be the same as they were in step s-1 and must be updated accordingly. If no neighbors were available in the current microfibril at z_s_, the enzyme would move to the nearest unoccupied lattice site at the new z-position.

The probabilities of displacement attempts were selected such that the enzymes would move with the diffusion coefficients reported in the literature. Collier et al. (2011) summarized the reported diffusion coefficients for collagenases and gelatinases on collagen and gelatin, highlighting the fact that the diffusion of the enzymes depends on whether or not the substrate is intact or cleaved (18). These experimental diffusion coefficients are summarized in Table 1. Because the diffusion coefficients for collagenase and gelatinase are not equal, the duration required for each displacement was selected such that each enzyme type would diffuse with its experimentally reported diffusion coefficient according to the relationship:

| $\left\langle{r(x,y,z)}^{2} \right\rangle=4Dt,$ | (9) |
| --- | --- |

where r is the displacement, x, y, and z are the coordinates of the enzyme found with Eq. 1-3, D is the diffusion coefficient, and t is duration of the step. The two-dimensional formulation of this equation was chosen because the enzymes are constrained to diffuse on the surface of the fibril.

#### MMP Cleavage Properties

Patterns of collagenase and gelatinase cleavage have been described previously (8, 9), and the model presented here was parameterized to mimic experimental observations. Collagenase cleavage only occurred when collagenases were positioned on the lattice site 66 nm from the C-terminus of an individual tropocollagen (i=4), and gelatinase cleavage only occurred when gelatinases were positioned on a tropocollagen that had already been cleaved by a collagenase (9, 19). When the enzymes were in the positions where cleavage could occur, cleavage succeeded at an overall rate of 0.35 s^-1^ (8). When cleavage occurred, a bond was broken between the N terminus of the occupied site and the C terminus of the site directly above the occupied site.

#### Collagen Fragment Removal

Once a lattice site and its lower neighbor had been cleaved, that site became disconnected from the rest of the lattice and was removed from the system immediately, unless otherwise specified. The removal probabilities were defined as

| $P\left( \mathrm{remove} \right)= \left\{ \begin{aligned} a, i, & i+1 cleaved \\ a, i=22, i-1 cleaved \\ a, i=1, i cleaved \\ 0, otherwise \end{aligned} \right.$ | (10) |
| --- | --- |
|  |  |

where a=1, except where otherwise specified.

Occasionally, a removed fragment was occupied by a gelatinase, which remained in its vertical position and was moved horizontally to an adjacent microfibril or the nearest available lattice site if no available sites existed on adjacent microfibrils.

As the removal progressed, some groups of lattice sites became detached from neighboring lattice sites and were removed from the system. A set of lattice sites was considered detached when it had no vertical neighbors, and when it was not attached to at least two other segments horizontally.
