# Supplementary material for: Effect of collagenase–gelatinase ratio on the mechanical properties of a collagen fibril: a combined Monte Carlo–molecular dynamics study
Source: Biomech Model Mechanobiol. 2019 Jun 3;18(6):1809–19. doi: 10.1007/s10237-019-01178-6 (PMC6825035; doi:10.1007/s10237-019-01178-6)
Supplement: Supplementary file 2 — Supplementary material 2 (DOCX 14 kb) [file 10237_2019_1178_MOESM2_ESM.docx]

#### S2 Text: Additional details on the Molecular Dynamics Model

#### Molecular Dynamics parameters

For the molecular dynamics simulations we used the parameters presented in S1 Table.

**a) Coarse grained model:** We implemented a collagen type I coarse grained, the parameters for the interaction potentials are given in Table 1. Tropocollagen is represented by a polymeric chain that contains 220 bonded beads units. Tropocollagen chains interact through a Lennard-Jones potential acting as the cohesive and repulsive force that keep the fibril together and prevent the interpenetration of beads, respectively. The potential is represented by the standard equation:

$U_{\mathrm{LJ}}=4\epsilon\left[ \left( \frac{\sigma}{r_{LJ}} \right)^{12}-\left( \frac{\sigma}{r_{LJ}} \right)^{6} \right]$ Eq.1

Where U_LJ_ is the potential energy due to the interaction between two tropocollagen beads i and j, r_LJ_ distance apart. The parameter ε represents the strength of the potential and σ is related to the position of the minimum in potential energy.

The bending angle between beads is controlled by a harmonic potential with the following equation:

$U_{\theta}=k_{\theta}\left( \theta-\theta_{0} \right)^{2}$ Eq.2

Where U_θ_ is the potential energy of the bending angle between three consecutively bonded tropocollagen beads i, j and k forming an angle θ. The parameter k_θ_ represent the bending strength in energy units and θ_0_ is the equilibrium angle.

The hyper-elastic bond (stretching) between tropocollagen beads is represented by 3-regime potential energy with a gradient defined by:

$F_{bond}=\frac{\partial U_{bond}}{\partial r}=\left\{ \begin{aligned} k_{T0}\left( r-r_{0} \right) if r<r_{1} \\ k_{T1}\left( r-r_{0} \right) if r_{1}\leq r<r_{break} \\ 0 if r>r_{break} \end{aligned} \right.$ Eq.3

Where r_0_ is the equilibrium distance between the two beads, k_T0_ and k_T1_ are the spring constants acting at different distances between 0 to r_1_ and r_1_ to r_break_, respectively. Note that the gradient of the potential energy represents the force of interactions between two given beads.

**b) Simulation parameters:** All the simulations were performed used LAMMPS molecular dynamics simulation package. The time step is 0.01 ps and the equations of motion are integrated with a Langevin thermostat that account for the implicit representation of water. The drag coefficient is 1000 ps and the temperature is 310K. A maximum velocity constraint is imposed to observe the breakage of the fibril without the collapse of the structure. The strains were applied in the axial direction with periodic boundary conditions. The strain rate used in these simulations was 10^7^ s-1. This strain rate represents a speed of 3.4 m/s for the dimensions of our fibril. Due to computational constraints the loading speed used in the current model was faster than strain rates employed in experimental paradigms.
