# Supplementary material for: Effect of collagenase–gelatinase ratio on the mechanical properties of a collagen fibril: a combined Monte Carlo–molecular dynamics study
Source: Biomech Model Mechanobiol. 2019 Jun 3;18(6):1809–19. doi: 10.1007/s10237-019-01178-6 (PMC6825035; doi:10.1007/s10237-019-01178-6)
Supplement: Supplementary file 3 — Supplementary material 3 (DOCX 13 kb) [file 10237_2019_1178_MOESM3_ESM.docx]

**S3 Text: Supplemental Results**

**Degradation Rate**

The fibril degradation rate was dictated by the total number of enzymes on the fibril, as well as the ratio of collagenases to gelatinases. By comparing the progression of degradation for 4 collagenases and 4 gelatinases (system 1) and 8 collagenases and 8 gelatinases (system 2), we see in Fig. 2 that the degradation progresses more rapidly when the number of MMPs is larger (system 2), as expected. However, when we compare systems with the same total number of enzymes (system 1, 3 and 4) we observe that the total amount of enzymes does not fully explain the rate of degradation. Rather, the ratio of collagenases to gelatinases appears to be the main contributor in the difference between systems. The rate of degradation increases as the ratio of collagenases to gelatinases decrease, this behavior is clearly observed in S1 Fig systems 1, 3 and 4.

S1 Fig also demonstrates semi-quantitative agreement between simulation results and experimental results for degradation, showing the mean $\pm$ s.d. for experimental measurements of degradation by Watanabe-Nakayama et al. {Watanabe-Nakayama, 2016 #288} along with the simulated results for the present study. However, notable differences between the experimental and simulated systems preclude a direct comparison of the degradation. First, the simulated results are for the combined action of fibroblast collagenase and gelatinase, while the experimental data are for degradation by bacterial collagenase, which has both collagenolytic and gelatinolytic action; the mechanism of degradation differs between experiments and simulations. Furthermore, the experimental results were obtained by varying the concentration of enzymes in the solution, while the simulations in the present work were obtained by varying the number of enzymes on the surface of the fibril, again precluding a direct comparison of our simulation results to the experimental results. For a discussion of the difficulties associated with quantifying the relationship between concentration of enzymes in solution and surface coverage of enzymes on the fibril, refer to the discussion section in the main text. Nevertheless, despite the differences between the experimental conditions and the conditions of our simulations, degradation in our simulations appears to progress in a manner consistent with the experimental observations.

Also, of note, System 2, with the larger number of MMPs, has a two-slope degradation behavior, with a first slow slope before 1 minute. This first slope is similar in value to the other systems, nevertheless the excess in enzymes seem to trigger an acceleration of the fibril degradation after 1 minute. Further analysis of this behavior will be necessary in future, though such analysis is beyond the scope of the present investigation.

S2 Fig shows directional motion of collagenase toward the N-terminus of the fibril, an emergent property of our simulations that provides further semi-quantitative verification of our degradation model. This directional motion has been observed experimentally in a number of different systems, though some disagreement exists about the direction of the motion along the fibril {Saffarian, 2004 #98;Sarkar, 2012 #102;Watanabe-Nakayama, 2016 #288}. Sarkar et al. (2012) and Saffarian et al. (2004) observed directional of MMP-1 along a line pointing from the N-terminus to the C-terminus, while Watanabe-Nakayama et al. (2016) observed motion along a line pointing from the C-terminus to the N-terminus. In the present work, the directional motion was in agreement with the latter with collagenases moving from the C-terminus toward the N-terminus. Furthermore, Saffarian et al. (2004) reported a flow velocity on the order of micrometers per second for MMP-1, which appears to agree semi-quantitatively with our simulation results presented in S2 Fig {Saffarian, 2004 #98}.
