# Supplementary figures and images for: Effect of collagenase–gelatinase ratio on the mechanical properties of a collagen fibril: a combined Monte Carlo–molecular dynamics study
Source: Biomech Model Mechanobiol. 2019 Jun 3;18(6):1809–19. doi: 10.1007/s10237-019-01178-6 (PMC6825035; doi:10.1007/s10237-019-01178-6)

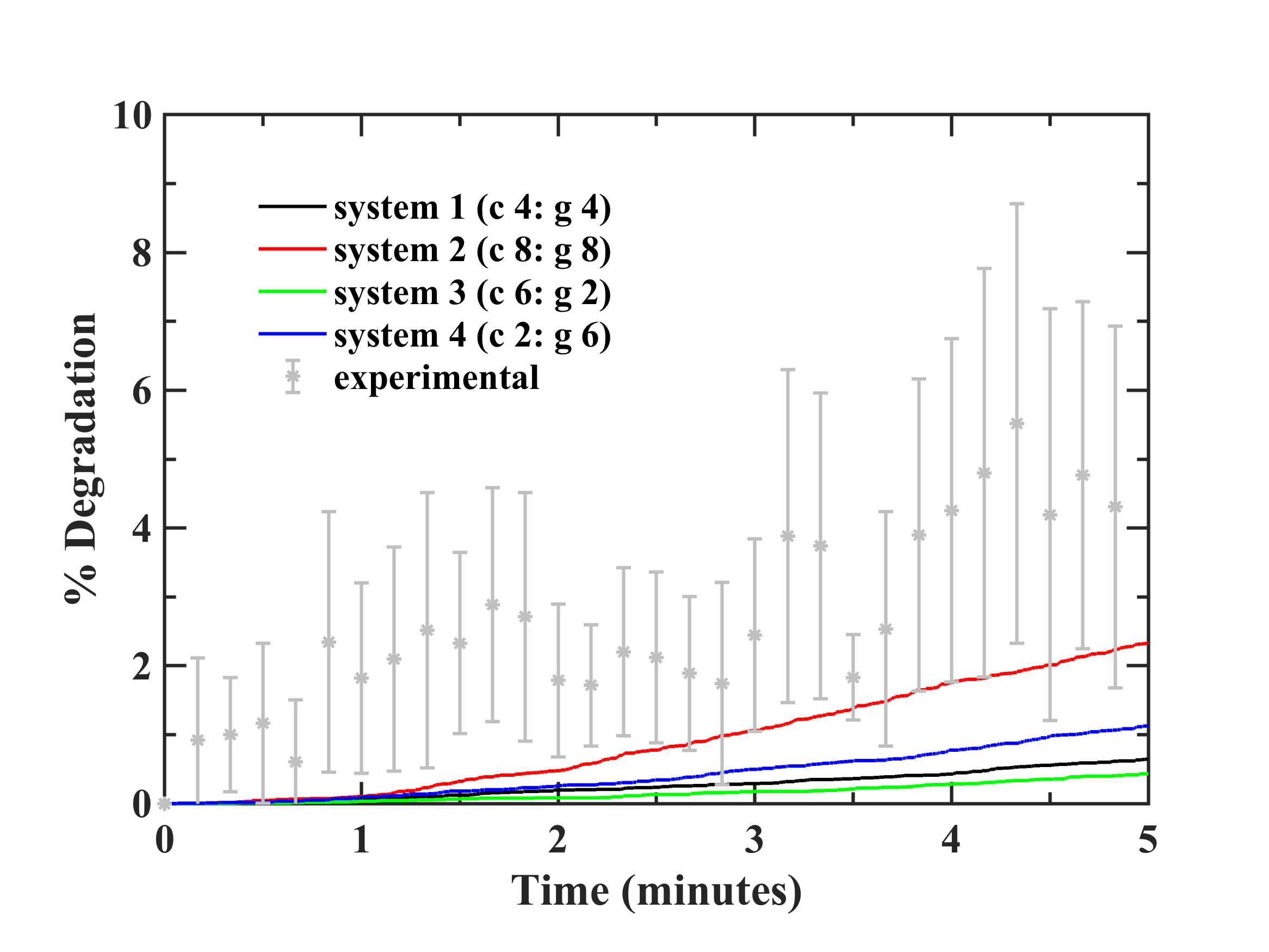

Supplement: Supplementary file 4 — S1 Fig. Degradation of collagen fibril by collagenases and gelatinases as a function of time for different collagenase to gelatinase ratios (TIFF 15952 kb) [file 10237_2019_1178_MOESM4_ESM.tiff]

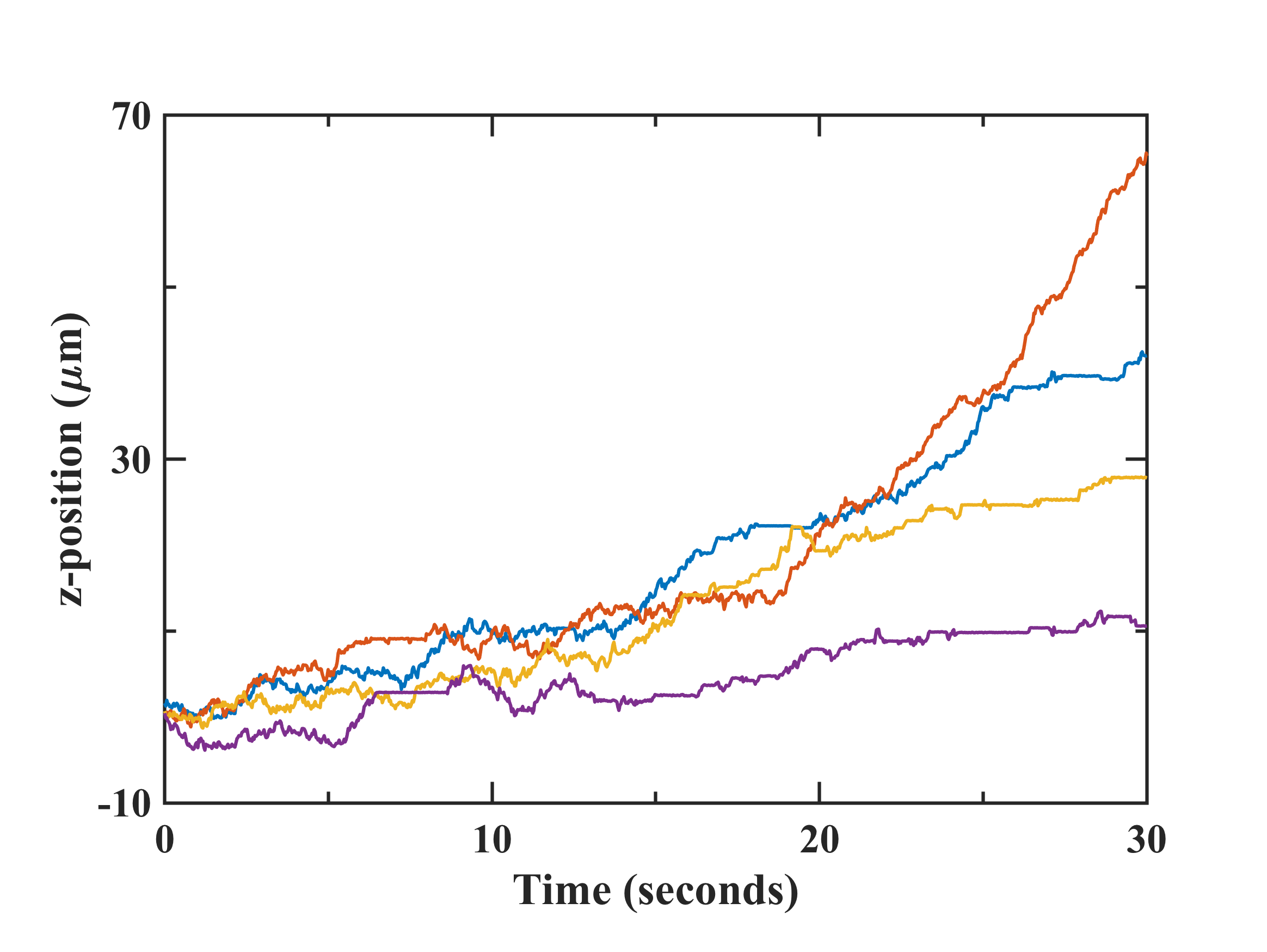

Supplement: Supplementary file 5 — S2 Fig. Trajectories for Collagenases Moving Along the Long Axis of the Collagen Fibril for a System with 10 Collagenases and No Gelatinases. Each curve represents the path of one of the five collagenases, showing the directional motion observed in our simulations. Though we did not include a bias toward directional motion a priori, we observed an emergent directional motion of collagenases from the C-terminus toward the N-terminus for all of the systems under investigation. z = 0 corresponds to the C-terminus of the fibril (TIFF 15909 kb) [file 10237_2019_1178_MOESM5_ESM.tiff]
