# Supplementary material for: Effect of collagenase–gelatinase ratio on the mechanical properties of a collagen fibril: a combined Monte Carlo–molecular dynamics study
Source: Biomech Model Mechanobiol. 2019 Jun 3;18(6):1809–19. doi: 10.1007/s10237-019-01178-6 (PMC6825035; doi:10.1007/s10237-019-01178-6)
Supplement: Supplementary file 6 — Supplementary material 6 (DOCX 14 kb) [file 10237_2019_1178_MOESM6_ESM.docx]

**S1 Table: Molecular Dynamics Model Parameters**

| Model parameters | Value |
| --- | --- |
| ε – Lennard Jones [Kcal/mol] | 6.87 |
| σ – Lennard Jones [Å] | 14.72 |
| θ_0_ – Equilibrium bending angle [degrees] | 180 |
| k_θ_ – Bending strength constant [Kcal/mol rad] | 14.98 |
| r_0_ – Equilibrium distance (tropocollagen) [Å] | 14.00 |
| r_1_ – Critical hypereslastic distance (tropocollagen) [Å] | 18.20 |
| r_break_ – Bond breaking distance (tropocollagen) [Å] | 21.00 |
| k_T0_ – Stretching strength constant (tropocollagen) [Å] | 17.13 |
| k_T1_– Stretching strength constant (tropocollagen) [Å] | 97.66 |
| r_0_ – Equilibrium distance (divalent crosslink) [Å] | 10.00 |
| r_1_ – Critical hypereslastic distance (divalent crosslink) [Å] | 12.00 |
| r_break_ – Bond breaking distance (divalent crosslink) [Å] | 14.68 |
| k_T0_ – Stretching strength constant (divalent crosslink) [Å] | 0.20 |
| k_T1_ – Stretching strength constant (divalent crosslink) [Å] | 41.84 |
| r_0_ – Equilibrium distance (trivalent crosslink) [Å] | 8.60 |
| r_1_ – Critical hypereslastic distance (trivalent crosslink) [Å] | 12.20 |
| r_break_ – Bond breaking distance (trivalent crosslink) [Å] | 14.89 |
| k_T0_ – Stretching strength constant (trivalent crosslink) [Å] | 0.20 |
| k_T1_ – Stretching strength constant (trivalent crosslink) [Å] | 54.60 |
| m – mass tropocollagen beads [a.m.u] | 1358.7 |
