# Supplementary material for: Effect of collagenase–gelatinase ratio on the mechanical properties of a collagen fibril: a combined Monte Carlo–molecular dynamics study
Source: Biomech Model Mechanobiol. 2019 Jun 3;18(6):1809–19. doi: 10.1007/s10237-019-01178-6 (PMC6825035; doi:10.1007/s10237-019-01178-6)
Supplement: Supplementary file 7 — Supplementary material 7 (DOCX 12 kb) [file 10237_2019_1178_MOESM7_ESM.docx]

**S2 Table:**

| Number of cleaved (but not removed) bonds at 1.1% degradation. | | | |
| --- | --- | --- | --- |
|  | Number of Collagenases | Number of Gelatinases | Cleaved Sites |
| System 1 | 4 | 4 | 700 |
| System 2 | 8 | 8 | 710 |
| System 3 | 6 | 2 | 999 |
| System 4 | 2 | 6 | 431 |
